# Gender equity in hiring: examining the effectiveness of a personality-based algorithm

**DOI:** 10.3389/fpsyg.2023.1219865

**Published:** 2023-08-15

**Authors:** Emeric Kubiak, Maria I. Efremova, Simon Baron, Keely J. Frasca

**Affiliations:** ^1^AssessFirst, Paris, France; ^2^King’s College London, Institute of Psychiatry, Psychology and Neuroscience, University of London, London, United Kingdom; ^3^Birkbeck Business School, Faculty of Business and Law, Birkbeck, University of London, London, United Kingdom

**Keywords:** gender, bias, algorithm, personality, hiring

## Abstract

**Introduction:**

Gender biases in hiring decisions remain an issue in the workplace. Also, current gender balancing techniques are scientifically poorly supported and lead to undesirable results, sometimes even contributing to activating stereotypes. While hiring algorithms could bring a solution, they are still often regarded as tools amplifying human prejudices. In this sense, talent specialists tend to prefer recommendations from experts, while candidates question the fairness of such tools, in particular, due to a lack of information and control over the standardized assessment. However, there is evidence that building algorithms based on data that is gender-blind, like personality - which has been shown to be mostly similar between genders, and is also predictive of performance, could help in reducing gender biases in hiring. The goal of this study was, therefore, to test the adverse impact of a personality-based algorithm across a large array of occupations.

**Method:**

The study analyzed 208 predictive models designed for 18 employers. These models were tested on a global sample of 273,293 potential candidates for each respective role.

**Results:**

Mean weighted impact ratios of 0.91 (Female-Male) and 0.90 (Male-Female) were observed. We found similar results when analyzing impact ratios for 21 different job categories.

**Discussion:**

Our results suggest that personality-based algorithms could help organizations screen candidates in the early stages of the selection process while mitigating the risks of gender discrimination.

## 1. Introduction

Research dating back as far as the 1970s (see Davison and Burke, 2000 for a review, which covers multiple countries) has shown that gender discrimination in hiring occurs, and continues to be a prevalent issue in today’s hiring practices–despite the findings from cross-temporal meta-analysis indicating that belief in competence equality has grown over time (Eagly et al., 2020). Yet, a recent meta-analysis of hiring discrimination experiments conducted between 2005 and 2020 (Lippens et al., 2023) reveals that gender discrimination is highly complex and varied, with instances of both males and females facing discrimination in certain cases. The relative advantages of male and female candidates hinge on demand-side factors. These may include the impact of certain job characteristics that are traditionally associated with one gender over the other on selection criteria, as well as how closely a candidate aligns with the typical characteristics of their gender category. Further substantiating these findings, a comprehensive meta-re-analysis of over 70 employment audit experiments conducted across more than 26 countries and five continents concluded that in male-dominated professions, which are typically higher-paying, being female can be a disadvantage. Conversely, in female-dominated professions, which tend to be lower-paying, being female is viewed positively (Galos and Coppock, 2023), thus confirming gender-role congruity bias. Besides, this bias consistently manifests in hiring decisions. For instance, Koch et al. (2015) meta-analysis concluded that it is more pronounced among male raters, and it does not diminish even when raters are provided with additional information about the candidate. However, despite both males and females facing discrimination based on occupation characteristics, the price paid by females is often higher than that of their male counterparts, as they often have limited access to higher-paying jobs and roles with greater responsibilities. In addition, in line with the backlash effect, which refers to a social and psychological phenomenon where individuals are penalized for violating societal norms or expectations regarding gender (Williams and Tiedens, 2016), females are in a double-bind. As concluded by Castaño et al. (2019) in a systematic review, “if women adopt masculine roles they are perceived as cold and instrumental, whereas if women adopt feminine roles they are perceived as less competent” (p. 0.14) –an effect that men do not typically experience. As a consequence, in highly prestigious occupations, even if females perform equally, they are rewarded significantly lower (Joshi et al., 2015). It results that the representation of females progressively declines higher up the hierarchy. Data from LinkedIn’s Economic Graph indicates that obstacles for females begin to appear as early as at the managerial level. Globally, only 25% of female ascend to the C-Suite level, even though the ratio of male to female is nearly equal at the individual contributor level (LinkedIn, 2022).

Addressing gender bias in hiring is not only a matter of ethical responsibility, but it is also crucial due to the harmful consequences such biases can engender. For example, a meta-analysis by Triana et al. (2019) found that perceived gender discrimination is negatively related to job attitudes, physical health outcomes and behaviors, psychological health, and work-related outcomes. Interestingly, even minimal biases can lead to substantial instances of hiring discrimination and losses in productivity, underscoring the significant practical impact of these biases. In a series of simulations, Hardy et al. (2022) established that a slight bias of 2.2% led to disparate treatment rates that were 13.5% higher than those observed in a bias-free model. Furthermore, the chances of a woman receiving a favorable hiring decision were almost halved, being 49% lower than the odds for their male counterparts with similar qualifications. The financial repercussions of this were significant, with bias accounting for 16.1% of new hire failure rates, ultimately leading to a utility loss per hiring due to bias amounting to -$710.54 per hire. The negative impact started to manifest with as little as 1% bias, which resulted in 8.7% disparate treatment and a utility loss of -$355.36. This effect escalated under the simulation of a higher 4% bias, where it led to 20.3% disparate treatment and a staggering utility loss of -$2,125.64. Furthermore, the authors found that contextual factors alter, but cannot obviate the consequences of biased evaluations. Consequently, it’s crucial to identify strategies that reduce bias in hiring decisions.

## 2. Intervention for mitigating gender bias

Efforts have been made to implement interventions for reducing gender discrimination, but yielded mixed outcomes. According to a recent systematic review, half of the intervention measuring social change in gender equality did not achieve beneficial results (Guthridge et al., 2022), leading the authors to conclude that “in the past 30 years we have not uncovered the keys to social change in order to enhance gender equality and non-discrimination against girls and women” (p. 0.335). In the specific context of recruitment, studies have consistently underlined the pervasive nature of unconscious stereotypes and the ease with which these biases can be triggered. For instance, Isaac et al. (2009), in a comprehensive review spanning over 30 years of research, found that conventional interventions, such as diversity training and employment equity programs, fail to guarantee gender equity in hiring despite their widespread use, and can even prove to be counterproductive. Similarly, counter-stereotype training appears to be effective only under specific conditions. Nevertheless, their review pinpointed several institutional interventions that could be promising to foster gender equity in hiring. They also highlighted actions that female applicants themselves could undertake. While their research suggests viable interventions to promote gender equity in hiring, it also underscores the issue’s complexity. Some recommendations appear as desperate “Hail Mary” attempts to combat gender bias. For instance, the advice, “If you are visibly pregnant, it might be wise to obscure it with your clothing” (p. 0.6), while effective, exposes the depth of societal bias we are grappling with. Also, it is important to recognize that such advice could be considered as pernicious, as it perpetuates and reinforces societal bias, rather than addressing the root causes of gender inequity in hiring practices.

Traditional interventions include diversity or counter-stereotype training (Bezrukova et al., 2012), the introduction of gender quotas (Krook and Zetterberg, 2014), lean-in approach (Chrobot-Mason et al., 2019), and equity guidelines. Yet, according to the International Labor Organization, even though 75 percent of companies worldwide have embraced policies of equal opportunity, diversity, and inclusion, gender biases stubbornly linger in selection (International Labour Organization [ILO], 2019). Indeed, despite good intentions, such interventions can have unintended consequences and potentially generate new issues. Caleo and Heilman (2019) synthesized the potential ways in which these interventions could backfire, including (1) promoting gender stereotyping, (2) reducing personal responsibility for bias, (3) fueling the perception of undeserved preference, (4) prompting negative trickle-down effects, (5) creating tokens, (6) encouraging discriminatory behavior, (7) depleting cognitive resources, and (8) doing harm to those who lead bias-reducing initiatives. Their analysis highlights the complex nature of gender bias in hiring, and the importance of carefully considering the unintended consequences of interventions. To this end, we’ll briefly explore the impact of conventional interventions in the next paragraphs.

Diversity training has been shown to be effective at reducing the extent to which assessors assign stereotypic labels to male candidates (e.g., determined and competitive) and female candidates (e.g., submissive and helpful) (Kawakami et al., 2007). However, this effect did not emerge if assessors engaged in the stereotyping task immediately after their counter-stereotype training. Despite the potential impact of training interventions, their effectiveness tends to be short-lived, with discrimination resurfacing as early as 3 months after the interventions (Derous et al., 2020). This finding emphasizes the limited long-term sustainability of such interventions in combating bias. Also, other research showed that training claiming to limit unconscious bias is ineffective, and ironically contributes to activating stereotypes (Madera and Hebl, 2013). For instance, Dobbin et al. (2007) conducted a study revealing that despite the adoption of sensitivity and diversity training by big corporations, there was no significant increase in gender diversity. This finding raises questions about the effectiveness of such training programs in achieving meaningful change. As argued by Noon (2018), unless the everyday discriminatory acts are effectively addressed, the adoption of such unconscious bias training in the workplace and in hiring may have limited utility to mitigate biases.

Gender quotas have been implemented as a means to address gender inequality, but research suggests that their effectiveness is not without drawbacks. While they aim to promote gender diversity, there are concerns that quotas may inadvertently reinforce stereotypes and fuel the perception that women are less competent. This is supported by findings from Leibbrandt et al. (2018), who found evidence of a severe backlash against women under gender quotas, leading to sabotage and undermining their success. Furthermore, the impact of quotas on corporate boards has also been examined. Yu and Madison (2021) conducted research showing that quotas for women on corporate boards have primarily resulted in decreased company performance. This raises questions about the direct correlation between quotas and improved outcomes. In addition, the introduction of gender quotas may intensify the negative effects of second-generation bias and perpetuate gender inequality in the workplace, as suggested by recent work by Loumpourdi (2023). This highlights the complex dynamics at play when implementing quotas and emphasizes the need for comprehensive approaches that address underlying biases and promote gender equality in a more nuanced and systemic manner.

Regarding equity guidelines, Ng and Wiesner (2007) found that implementing basic employment equity messages only had a positive impact when underrepresented group members were equally or more qualified than the majority group. However, when preferential treatment was given to less qualified candidates, men who were underrepresented in the profession tended to be favored over underrepresented women. Similarly, stronger employment directives typically led to detrimental outcomes whereby such perceived coercive employment equity messages resulted in men being favored over women. As a result, Castilla and Benard (2010) draws a paradoxical conclusion that in organizations that strongly advocate for meritocracy, decision-makers tend to exhibit a preference for men over equally qualified female employees. This finding highlights a discrepancy between the professed value of meritocracy and the actual biases that can influence decision-making processes.

Considering the evidence of gender biases in selection and assessment, in conjunction with interventions that are shown to be largely ineffective, there is a need to explore alternative ways of mitigating these issues. This is particularly important since gender diversity is positively related with higher employee wellbeing and positive job appraisal (Clark et al., 2021), as well as with productivity in contexts where gender diversity is viewed as normatively accepted (Zhang, 2020). To address these challenges, an important step is to enhance the structure of the evaluation and selection procedure. For example, Wolgast et al. (2017) showed that using tools for systematizing information about the applicants could help in mitigating biases and in selecting more competent applicants.

## 3. Algorithms as a solution against bias

One solution to alleviate biases could be the use of hiring algorithms, which allow us to go beyond our intuition and cognitive biases, by bringing standardization and structure to hiring decisions. An algorithm could be defined as a set of operations or tasks to be carried out following a certain logic, with the aim of answering a question or solving a problem (Jean, 2019). In other words, an algorithm acts like a set of instructions, turning the information we feed into it into recommendations. The utilization of algorithms at different steps of the hiring pipeline is becoming increasingly prevalent in today’s workplace (Tambe et al., 2019), and systematic review point out the potential for these algorithms to revolutionize HR management (França et al., 2023). In the hiring process, algorithms learn from past data of old applicants to predict how suitable future applicants might be for a job. Basically, they figure out what attributes from past successful applicants led to good job performance, and then use this understanding to predict which future applicants might be the best fit for the job. The increasing adoption of algorithms is largely driven by their efficiency. For example, in a meta-analysis comparing mechanical and clinical data combination in selection, Kuncel et al. (2013) showed that, in predicting job performance, the difference in the validity between mechanical and clinical data combination methods resulted in an enhancement of prediction accuracy exceeding 50%. Other studies showed that algorithms make better hiring decisions in terms of the employee’s performance outcomes (Sajjadiani et al., 2019; Li et al., 2020) and in hiring fill rate (Horton, 2017). Taken together in a recent systematic literature review, these results regarding performance suggest that algorithmic hiring methods are equal or better than human when selecting the best candidates (Will et al., 2022).

Recently, scholars have also been advocating for the use of such algorithms to reduce implicit biases in hiring processes and have proposed frameworks to evaluate AI-assisted interventions (Lin et al., 2021). According to Leutner et al. (2022), by using AI for hiring purposes, employers will be able to control for not only gender bias but other discriminatory characteristics as AI technology is able to be trained in a way to filter through the necessary characteristics required for candidates and to ignore other features. This is supported by several studies, showing that machine learning has no adverse impact on gender (Sajjadiani et al., 2019) or that a fair ranking algorithm could increase the selection of female candidates (Sühr et al., 2020). In regards to empirical evidence, Li et al. (2020) revealed that some algorithms could increase the share of women selected, up to a balance of 50%, compared to 35% for hiring decisions made by humans. Similarly, Avery et al. (2023) conducted a comparative analysis between human-evaluation and AI-evaluation treatments. The authors found that human evaluators consistently rated males higher than females by a substantial 0.15 standard deviations. This gender discrepancy was most noticeable at the higher end of the distribution, with men being 6.8 percentage points more likely to rank in the top 25%, and 7.73 percentage points more likely to land in the top 10%. In contrast, when AI was employed, the gender difference shrank considerably to just a 0.04 standardized difference. Furthermore, the representation of males and females in the top 50%, 25%, and 10% categories under the AI condition was nearly equal, showcasing the potential for AI to mitigate human biases in evaluation processes. Hiring algorithms could also benefit by increasing the perceived equity of the hiring process. For example, (1) women prefer to be judged by an algorithm because of its perceived objectivity over a human (Pethig and Kroenung, 2023), (2) algorithms are perceived as less discriminatory than humans, which increases people’s comfort toward their usage (Jago and Laurin, 2021), and (3) applicants with prior discrimination experiences deem algorithm-based decisions more positively than those without such experiences (Koch-Bayram et al., 2023).

Despite these findings, many researchers have sounded the alarm. For instance, Drage and Mackereth (2022), in their review of assertions made by AI providers, suggested that endeavors to eradicate gender and race from AI frequently misinterpret these concepts as discrete characteristics rather than broader structures of power, or that that using AI as a fix for gender diversity issues, an example of technosolutionism, fails to address the inherent systemic issues within organizations. Others raise concerns that algorithms could unintentionally exacerbate existing biases within recruitment processes (Kelly-Lyth, 2021). Algorithmic bias takes on a discriminatory aspect when it results in consistent disparities linked to factors legally protected, such as gender. For instance, Dastin (2022) documented a case involving Amazon’s hiring algorithm, which persistently gave higher employability scores to men than to women, while Chen et al. (2018), testing the adverse effects of candidates search engines, showed that female candidates were ranked statistically lower than male candidates. This circumstance has prompted scholars to delve into the exploration of algorithmic biases in hiring and strategies to mitigate them (De Cremer and De Schutter, 2021). From a psychological perspective, research shows that, while individuals view algorithm-driven decisions as less prone to bias, they also generally regard it as less fair (Feldkamp et al., 2023). Moreover, algorithmic decisions resulting in gender disparities are less likely to be perceived as biased compared to human decisions, because people tend to believe that algorithms make decisions devoid of context, thereby disregarding individual characteristics (Bonezzi and Ostinelli, 2021). From a technical perspective, Rieskamp et al. (2023) identified four types of strategies aimed at reducing discrimination in these systems, namely pre-process, in-process, post-process, and feature selection. This review implies that interventions can be implemented at various stages of the algorithm development process to effectively mitigate bias. This is supported by van Giffen et al. (2022), who listed different types of biases in algorithm and in machine learning, distinguishing, for example, biases related to the use of historical biased data (Mehrabi et al., 2019), data which are not representative for the relevant population, or measurement biases. However, intervening to reduce subgroup differences in selection often presents a trade-off regarding accuracy. This situation represents what is known as the validity-diversity dilemma, which involves maintaining a balance between selecting valid performance predictors and minimizing adverse impact. While interventions aimed at reducing subgroup disparities could decrease model accuracy (Zhang et al., 2023), strategies employing multi-penalty optimization are promising in addressing this issue (Rottman et al., 2023).

In summary, these findings suggest that training an algorithm to predict the preferences of a recruiter and mimic human intuition will inevitably surface and amplify biases. On the other hand, training an algorithm to predict genuine success, using more gender-blind data that accurately forecast job performance, will likely mitigate biases in hiring decisions. This understanding underpins the guidelines on AI-Based Employee Selection Assessments provided by the Society for Industrial and Organizational Psychology (SIOP). The organization strongly urges providers to generate scores that (1) are considered fair and unbiased, (2) are clearly related to the job, (3) predict future job performance, (4) produce consistent scores that measure job-related characteristics, and (5) documented for verification and auditing (Society for Industrial and Organizational Psychology [SIOP], 2023). In other words, “from both research and workplace law perspectives, a clear and theoretically founded link should be established between the outcome (e.g., predicted job performance) and the algorithmic features” (Society for Industrial and Organizational Psychology [SIOP], 2020).

Considering these guidelines, there is great potential for using algorithms to reduce gender discrimination in hiring if it is personality-focused since theory proposes that personality predicts job performance (Schmitt, 2014), and does not vastly differ between genders. For instance, the gender similarities hypothesis (Hyde, 2005) suggests that males and females are similar in most psychological variables. With respect to empirical evidence, when personality facets are examined separately, the effect sizes are close to zero in most cases (Zell et al., 2015). Still, other scholars suggested that some differences between genders exist, with the most impacted facets being those related to agreeableness and neuroticism (Weisberg et al., 2011; Kajonius and Johnson, 2018). Thus, the extent of gender differences observed in research findings is still a subject of debate among scientists: some argue that these findings are more commonly characterized by similarities, while others assert that substantial differences are frequently observed. Interestingly, new findings show that gender differences or similarities are reflecting differing ways of organizing the same data, leading Eagly and Revelle (2022) to recommend “recognizing the forest and the trees of sex/gender differences and similarities. It is necessary to step away from the individual trees, perhaps to a hilltop, to observe the patterning of trees in a forest” (p. 1355). While minor differences may exist on particular facets, it is, therefore, essential to transcend a one-dimensional understanding and view the broader picture, observing how the aggregation of various personality facets can highlight distinct differences between genders, or potentially offset certain differences observed within a single facet. For example, while larger differences emerge from averaging multiple indicators that differ by gender (Eagly and Revelle, 2022), one could expect that such differences will be lowered by aggregating a facet that differs by gender with others that do not. Contextualizing the measure of personality (Judge and Zapata, 2015), in order to benefit from the information brought by facet-level (Soto and John, 2017), as well as reducing adverse impact, is, therefore, an intriguing path to explore.

More precisely, it is interesting to look at whether or not personality facets aggregates will lead to bias and adverse impact in personality-based hiring algorithms. Recent research has examined the accuracy of personality prediction in AI-based hiring systems and found that certain tools demonstrate significant instability in measuring key facets. Consequently, these tools cannot be considered valid assessment instruments (Rhea et al., 2022). However, it is still uncertain whether alternative personality-based hiring algorithms, designed to predict job performance based on personality facets, could potentially result in adverse impacts or biases. Indeed, training an algorithm based on personality data, and teaching it to identify relevant and non-gendered cues of performance for a role, could probably help (1) in hiring people who perform better, as personality is predictive of job performance (Schmitt, 2014) and who turnover less (Kubiak et al., 2023b), and in (2) achieving natural gender balance for different roles, because even though differences in specific personality facets between genders exist, these differences are smaller compared to other attributes currently used in hiring decisions (Kuhn and Wolter, 2022). Initial findings provide support for this hypothesis, demonstrating that specific personality-based algorithms exhibit gender fairness (Kubiak et al., 2023a). However, these studies were limited in their scope and examined a small number of roles.

Therefore, our study introduces a new breed of algorithms for multiple reasons. Firstly, it employs a personality-centric approach, which stands in stark contrast to conventional algorithms that aim to digitize existing hiring procedures by training on data from candidates’ resumes. Such data is riddled with bias (Parasurama et al., 2022), which inevitably trickles down into the algorithmic results (Houser, 2019). Secondly, our algorithm strives to predict future job performance, a marked departure from other algorithms that merely assess personality without making job performance projections. Thus, our study’s algorithms primarily target the identification of personality aspects that drive job performance in a specific occupation, subsequently scoring candidates by juxtaposing their personality, gauged through a personality assessment, against these predictive factors. Finally, to counteract the often-criticized “black box” effect (Ajunwa, 2020), our algorithms are based on explainable regression methods, in order to ensure efficiency but also transparency of the operations.

Our study expands the current knowledge, with the objective of testing whether we can adopt a personality-based algorithm to make hiring recommendations, whilst eliminating any adverse impact with regards to gender. Therefore, we hypothesize that a personality-based hiring algorithm would recommend hiring female and male candidates in (almost) similar proportions for different roles.

## 4. Materials and methods

This study involved the use of diverse samples. Firstly, training samples were utilized to construct predictive models for each occupation. Predictive modeling, as defined by Kuhn and Johnson (2013), is “a process of developing a mathematical tool or a model that generates an accurate prediction” (p. 2). In our study, a predictive model is defined as a combination of personality facets that generates an accurate prediction of job performance for a specific occupation. Secondly, a global analysis sample was employed to evaluate any potential adverse impact. For the sake of convenience, these samples will be referred to as training samples and analysis sample in the following sections.

### 4.1. Participants

Training samples were based on data from 18 employers, all clients of a specialized online assessment platform called “AssessFirst,” dedicated to predictive hiring and personality assessments. These employers specialized in different industries, including retail, technology, consulting, finance and banking, engineering or transportation. Furthermore, the size of the companies varied significantly within the selected group. The range included small-sized companies with approximately 100 employees, as well as large international corporations with over 50,000 employees. Most were located in France (39.90%), followed by Russia (22.60%), the USA (13.46%), and the United Kingdom (9.62%). Other countries included Brazil, Austria, Chile, Germany, Hungary, Morocco, Poland, Portugal, Romania, South Africa and Ukraine. These countries provided a broad geographical base that further enhanced the generalization of the results. These employers were using the platform in a high-stake hiring context, in order to enhance their selection and assessment processes with a heightened degree of objectivity. By using the online recruitment platform, these organizations endeavored to refine their hiring practices. They utilized the platform’s capabilities to construct predictive models for the occupations they sought to fill. The process for developing predictive models is described in the next section. This approach facilitated the comparison of prospective candidates’ personality profiles against the established predictive model, providing a comprehensive analysis of how well a candidate’s personality aligns with the specific requirements of the occupation. This thorough evaluation offered them deep insights, enabling them to make well-informed and objective hiring decisions. The selection of employers for this study was based on their active usage of the online platform during the period between 2021 and 2022. The primary criterion for inclusion was their utilization of the algorithmic-driven predictive model generation feature offered by the online platform. We only integrated into the samples employers who have undergone extensive training on platform usage and have demonstrated their proficiency by creating multiple predictive models. This stringent approach guaranteed that employers who were part of the sample were utilizing the platform correctly. The selection process focused solely on these aspects, without any commercial considerations involved. The purpose of this sampling approach was to ensure that the employers chosen had experience with and utilized the specific feature being investigated, allowing for targeted analysis of the predictive models generated through the platform.

### 4.2. Models generation

Our study hinged on data provided by these 18 employers, involving 208 unique occupations they were recruiting for. A total of 21 job categories were represented, predominantly sales (26.92%), financial services (13.46%), customer service (10.58%) and business development (7.69%). For each occupation, a distinct predictive model was designed, totaling 208 predictive models. In our study, we focused on developing predictive models that specifically considered the personality facets relevant to job performance in a given occupation. For example, Company 1, which was recruiting for a human resource role in Hungary, generated a predictive model that incorporated the personality facets of Extraversion, Agreeableness, and Openness. These facets were selected by the algorithm based on their statistical associations with job performance in that particular role. It is important to highlight that our algorithm exclusively relies on personality-related data (scores ranging from 1 to 10 on 20 personality facets) and performance-related data (scores ranging from 1 to 5). Our approach represents a departure from traditional hiring algorithms, which typically rely solely on data extracted from the CV or resume of candidates. Instead, we introduced a novel methodology that goes beyond CV data and incorporates personality facets relevant to job performance. Predictive models were generated directly by the employers using a dedicated feature on the online platform. The online platform provider describes the feature as an algorithm-based contact analysis tool that empowers employers to autonomously analyze their data and generate data-driven predictive models for the specific occupations they are hiring for. This tool leverages algorithms to extract insights from the data provided by employers, allowing them to uncover valuable patterns and relationships between personality facets and job performance. The process of predictive model creation in our study was, therefore, characterized by two distinct data collection stages. This was subsequently followed by the application of an algorithm, which selected the relevant personality facets to predict performance in the role. This approach ensured a well-rounded, scientific basis for all the predictive models devised in the study. The process of predictive modeling in the online platform works as follows:

– First, employers selected a representative sample of current employees in the occupation they were recruiting for. For instance, Company 1 chose a sample of 20 employees in the Human Resources role. To accomplish this, employers simply sent invitation emails to the selected employees through the online platform. In this study, it is important to note that the authors did not have direct contact with the employees involved. Instead, the employees were invited by their respective employers to participate in the study. The responsibility of explaining the purpose of the invitation to the selected employees rested with the employers. Subsequently, each employee independently created an account on the platform and provided their consent for their data to be utilized by the employer specifically for the purpose of predictive modeling. Once their account on the online platform was created, employees were asked to complete a forced-choice personality questionnaire. On average, it took approximately 12 min to complete the questionnaire, which consisted of 90 items. The personality questionnaire utilized a hierarchical model of personality based on the Five-Factor Model (FFM). It assessed 20 facets, with each personality trait being evaluated through four distinct facets. The scoring of each facet was done using Item Response Theory (IRT) modeling, and calibrated on a scale from 1 to 10, according to a Gaussian distribution. Following the completion of the assessment, each employee was, therefore, positioned and evaluated in terms of the 20 personality facets. This positioning allows us to understand and quantify the individual’s characteristics and tendencies across the various personality facets. Extensive research has demonstrated the questionnaire’s strong predictive validity ($\bar{x}$ = 0.63), as well as its reliability, as measured by Cronbach’s alpha (α = 0.79) and test–retest reliability (*r* = 0.80). Additionally, the questionnaire exhibits high sensitivity (δ = 0.96). The number of employees across the 208 training samples of our study varied from 20 employees to 151 (*M* = 41).
– Secondly, the performance of each employee was assessed by their respective direct manager. Managers autonomously accessed the online platform and assigned a rating to each employee within their respective training sample using a standardized scale ranging from 1 (indicating very poor performance) to 5 (reflecting excellent performance). A standardized scale was privileged to ensure objectivity, consistency, comparability and easiness of data collection. During the rating process, managers were prompted to consider the employee’s proficiency and objective job performance, such as revenue generation in the case of a sales occupation. To ensure accuracy of the performance ratings, definitions for each score were directly proposed within the online platform as guidance. This allowed for a comprehensive evaluation of each employee’s performance based on the manager’s insights and observations.
– Thirdly, once all employees in the training sample for one of the 208 occupations had completed the personality assessment and received ratings from their managers, the online platform automatically employed regression-based algorithms to analyze the data. Regression analysis is a statistical technique used to model the relationship between a dependent variable and one or more independent variables. This type of algorithm was chosen by the online platform because it is considered a fundamental algorithm in machine learning and artificial intelligence (Hope, 2020), in particular due to their: (1) simplicity, as regression is straightforward to understand and interpret, (2) efficiency, as regression is computationally efficient compared to other algorithms, and does not require heavy computational resources, and (3) predictive insights, as regression provides coefficients for each feature, indicating the relationship between the feature and the output variable, thus allowing for a high degree of explainability. In the context of our research, regression-based algorithms were employed to uncover patterns and relationships between the personality facets assessed through the questionnaire and the performance ratings provided by managers. These algorithms use the collected data to determine the extent to which each personality facet influences performance. The process involves fitting a regression model to the data, which estimates the coefficients or weights associated with each personality facet. These coefficients quantify the strength and direction of the relationship between the independent variables (personality facets) and the dependent variable (performance rating). The regression algorithm learns from the data and iteratively adjusts these coefficients to minimize the difference between the predicted performance and the actual performance ratings. By employing regression algorithms, the online platform automatically analyzes the data, identifies the most influential facets, and constructs a predictive model specific to the occupation. This model can then be used to predict candidates’ future performance. Therefore, while regression analysis itself is a statistical technique, when utilized within an automated system or platform to analyze data and make predictions, it can be considered part of the broader field of algorithms and machine learning (Hastie et al., 2009; Goodfellow et al., 2016). It is worth noting that prior to deployment, the validity of each predictive model (*N* = 208) was estimated and showed good accuracy, precision, recall and ROC AUC. Results are presented in Table 1.

**TABLE 1:** Summary of validity metrics of predictive models (*N* = 208).

Metric	*M*	*SD*
Accuracy	0.82	0.10
Recall	0.85	0.12
Precision	0.91	0.11
ROC AUC	0.80	0.14

The predictive models used in this study were autonomously created by individual employers. All relevant data pertaining to each model, including the training sample used, performance score, and results of the regression analysis (i.e., the facets taken into account in the models and score’s expectation for each), were securely stored in the database, following GDPR regulations, of the online recruitment platform. This database served as a repository for the information related to the predictive models created by each employer and was re-used for the purpose of this study.

### 4.3. Procedure

The initial step of the procedure involved collecting data related to the 208 predictive models from the database. During this process, no filters or selection criteria other than previously mentioned were applied, and all the predictive models created by trained clients of the online recruitment platform between 2021 and 2022 were included. This approach ensured that a comprehensive dataset was obtained, encompassing all available models within the specified timeframe, without any exclusion or bias in the selection process. Author 1 and 3, being affiliated with the online recruitment platform, had convenient access to facilitate the data collection process. For each predictive model, a dataframe consisting of various variables was available. These variables encompassed the following information: (1) name of the company, (2) job category associated with the occupation, (3) specific occupation name, (4) country, (5) data regarding the users included in the training sample, including scores for each personality facet and performance score, in a JSON format (6) facets incorporated in the predictive model and the score expected on each facet, either a high score or a low score, and (7) performance metrics of the predictive model, including accuracy, recall, precision, and ROC AUC.

The second step of the procedure was to assess the potential adverse impact of the predictive models created regarding gender. For this, we constituted a global analysis sample of “potential candidates” who had already taken the personality assessment and had profiles on the online recruitment platform was utilized. These participants have registered on the platform at different times, motivated by various reasons such as receiving invitations from companies or simply wanting to explore and learn more about themselves through the assessments. This approach was chosen for several reasons: (1) utilizing the existing pool of individuals who already had profiles and had taken the personality assessment on the online platform allowed for convenient access to a substantial sample size, (2) although these individuals may not have applied to one of the 208 specific occupations studied, they represented a global population of individuals who could potentially apply for those occupations, broadening the scope of the analysis, and (3) by utilizing this approach, we were able to explore a wider range of predictive models compared to the limitations imposed by using real candidates or specific samples for all 208 occupations. By adopting this methodology on a global scale, we were able to successfully conduct this study on a large and diverse participant pool. The testing sample, therefore, consisted of individuals who met the following criteria: (1) created an account on the online assessment platform in 2022, (2) completed the same personality assessment as described earlier, and (3) provided consent for their anonymized data to be used for scientific and publication purposes. In this specific research, individuals were not directly informed or contacted. However, their data was used with their consent once they registered on the online platform. The analysis sample comprised 273,293 individuals, with 51% identifying as females and 49% as males. The majority of the sample was primarily from France (*n* = 210,364, 77%) and held either a master’s degree (*n* = 97,584, 36%) or a bachelor’s degree (*n* = 96,405, 35%). The study involved access to specific information for each individual in the analysis sample. The available information included the following data points: (1) a score ranging from 1 to 10, representing the measurement of 20 personality facets through the use of a personality assessment employed in this research, and (2) the gender of the individual, categorized as either male or female. By utilizing this global sample, the methodology aimed to assess the potential impact of gender, providing a comprehensive understanding of how males and females scored in relation to each predictive model and the corresponding recommendations made by the scoring algorithm. For each individual within the analysis sample, a fit score was calculated, representing the level of alignment between their personality profile and the predictive model utilized. The fit score were ranging from 0 to 100%. For the purpose of the analysis, this fit score was calculated in a simple way. If the score of the candidate on a facet aligns with the expectation of the predictive model, the candidate was attributed a maximum score for the facet. If the score of the candidate on a facet is opposite to the expectation in the predictive model, the candidate was attributed a minimum score for the facet. If the score of the candidate on a facet taken into account is neutral, the candidate was attributed a medium score for the facet. Then, a simple formula calculates the fit score by summing the individual facet scores and dividing it by the total number of facets in the predictive model. The total result is then multiplied by 100 to express it as a percentage. A higher fit score, closer to 100%, indicated a stronger alignment between the candidate’s profile and the facets that explained performance in the occupation, suggesting a higher likelihood of success on the role. Overall, individuals in the analysis sample who scored above 60% or above the 70th percentile on a predictive model were considered as recommended candidates by the online recruitment platform. Others who fell below these thresholds were not considered recommended. The choice of this threshold was based on studies conducted by the online platform, which demonstrated that individuals scoring above the 70th percentile had better performance and retention rates in the months following their hiring (Kubiak et al., 2023b). Following this procedure, we obtained the fit scores of the 273,293 individuals composing the analysis sample for each of the 208 predictive models. The average fit score for females was 52.97 (*SD* = 11.48), and 53.22 for males (*SD* = 11.56).

### 4.4. Analysis

To analyze fairness and adverse impact, we applied the concept of impact ratio. The impact ratio is a statistical measure used to assess adverse impact in employment practices, particularly in the context of equal employment opportunity and fair hiring practices. It generally compares the selection rate of a protected group to the selection rate of a reference group (typically the group with the highest selection rate) within a specific job or employment process. The impact ratio is calculated by dividing the selection rate of the protected group by the selection rate of the reference group. In our first analysis, the impact ratio was calculated by dividing the recommendation rate for females (proportion of females who scored above 70th percentile or fit score above 60%) by the recommendation rate for males. Instead of using the selection rate as the metric, the recommendation rate was chosen for evaluation. This decision was made because the algorithm functions as a tool to provide recommendations to employers, rather than making independent decisions. The clients utilizing the online platform are the ultimate decision-makers. Therefore, in order to assess the fairness of the algorithm’s recommendations, rather than the fairness of human decisions, the recommendation rate was deemed more relevant for the research’s objective. While the focus of the analysis was on females as the protected class, considering evidence of discrimination against them, a reverse analysis was also conducted with males as the protected class. This allowed for a comprehensive evaluation of fairness across both genders. Guidelines from the Equal Employment Opportunity Commission (U.S. Equal Employment Opportunity Commission [EEOC], 1979), specifically the four-fifths rule, were followed to assess fairness. According to the rule, a selection tool, or a predictive model in the context of our study, with an impact ratio between 0.8 and 1.0 is generally considered fair. Impact ratios below the threshold of 0.8 indicate a disparate impact, meaning the algorithm or selection method tends to recommend more of one gender over the other. The impact ratios were examined using two approaches: (1) mean weighted impact ratio across all predictive models, and (2) impact ratios broken down by job category, providing a detailed analysis of fairness in each specific job category. By considering these measures, the study aimed to evaluate the fairness of the predictive models and identify any potential disparities in recommendation rates between genders, in accordance with EEOC standards.

## 5. Results

In the first analysis, which considered females as the protected class when calculating the impact ratio (Female-Male), we identified 124 predictive models with impact ratios ranging from 0.74 to 1 (mean weighted impact ratio = 0.91; *SD* = 0.06). The remaining 84 models had impact ratios higher than 1, and were, therefore, considered in the second analysis, with males as the protected class. It is worth mentioning that only eight models from the 124 had impact ratios below 0.8, and were really close to the threshold defined by EEOC. Also, Cohen’s d showed no significant effect on average (mean |*d*| = 0.11). Results of analysis 1 are presented in Table 2. In the second analysis (Male-Female), as expected, 84 models were identified, with impact ratios ranging from 0.71 to 0.99 (mean weighted impact ratio = 0.90; *SD* = 0.06). Only 3 models missed the 0.8 threshold, and Cohen’s d showed no significant effect on average (mean |*d*| = 0.11). Results of analysis 2 are presented in Table 3.

**TABLE 2:** Summary of results for analysis 1 (female-male; *N* = 124).

Metric	*M*	*SD*
Impact ratio	0.91	0.06
Female fit	0.52	0.11
Male fit	0.53	0.11
Cohen’s *d*	0.11	–

**TABLE 3:** Summary of results for analysis 2 (male-female; *N* = 84).

Metric	*M*	*SD*
Impact ratio	0.90	0.06
Female fit	0.54	0.12
Male fit	0.53	0.12
Cohen’s *d*	0.11	–

To examine potential impact further, we examined the average impact ratio by job category. Results are presented in Table 4 and show that the average impact ratios are above the 0.8 threshold for every category. The lowest results were for the categories “human resources” and “management board” when males were considered as the minority group, with mean weighted impact ratios of 0.82. Even so, this is in line with EECO standards and supports our study hypothesis.

**TABLE 4:** Mean impact ratios by job category (*N* = 208).

Job category	*N*	Analysis 1 (female-male)	Analysis 2 (male-female)
Accounting/auditing	5	0.99	0.91
Administrative	10	0.94	0.92
Business development	16	0.94	0.92
Computer	1	0.95	–
Consulting	13	0.89	0.84
Customer service	22	0.92	0.88
Design/creative	2	0.85	0.95
Education	1	0.98	–
Entrepreneurship	2	0.90	0.88
Financial analyst	28	0.90	0.89
Human resources	5	0.86	0.82
Management/board	12	0.91	0.82
Marketing	2	0.96	0.93
Media/communication	1	0.80	–
Military	3	0.90	0.88
Product management	1	–	0.91
Purchasing	10	0.94	0.91
Real estate	3	–	0.91
Sales	56	0.91	0.92
Tech/data	7	0.96	0.87
Other	8	0.93	0.89

Also, to simulate and test each predictive model, we chose to test them on a neutral sample composed of so-called “potential candidates” who were people derived from a global population. In practice, however, candidates who will be scored by the algorithm have higher chances of holding a similar and specific position, which is related to the predictive model (e.g., salespeople for a sales representative predictive model). We ran a preliminary analysis to estimate how testing the algorithm on a specific sample would impact the results. This analysis was conducted on three different occupations: project manager, customer service representative and technician. Overall, impact ratios did not differ significantly and were still matching the EEOC requirements. Results are presented in Table 5. While promising, these results were obtained through analyzing three jobs only, and further investigation at a larger scale is required to ensure that results replicate with specific samples.

**TABLE 5:** Comparison of impact ratios depending on the type of sample.

Occupation	Impact ratio	Cohen’s *d*
Project manager	0.96 *(0.95)*	0.02 *(0.02)*
Customer representative	0.93 *(0.87)*	0.07 *(0.12)*
Technician	1.14 *(0.83)*	−0.05 *(0.09)*

Results in brackets are those observed using a global sample.

## 6. Discussion

This research focus stemmed from alarmingly high gender discrimination that is ongoing in selection and assessment, despite legislation that should prevent discrimination on the basis of gender. To overcome such biases and improve selection, recent years have seen an increase in the use of algorithms in hiring decisions. Nevertheless, little is known about how these kinds of algorithms are used in practice, and some vendors of algorithmic pre-employment assessments are too opaque about the fairness of their solution (Raghavan et al., 2020). Also, while these systems are increasingly subject to technical audits regarding their performance, there is still a lack of proof to support the claims being made by such tools (Sloane et al., 2022). Still, new evidence has shown that using hiring algorithms could help in making better hiring and reducing human bias in selection (Lakkaraju et al., 2017; Li et al., 2020; Will et al., 2022). These examples should not, however, hide other widely publicized and criticized practices, where the use of algorithms has contributed to exacerbating gender discrimination. Instead, it must open the way to the development and usage of more ethical algorithms, where the beneficial effects prevail. To address this issue, one must rely on data which are mostly gender-blind and are truly predictive of performance. Even if they are widely used in current hiring algorithms, pieces of information from the CV do not meet this double requirement, and force the reproduction of gender bias in selection. There is, indeed, a lot of gendered data in someone’s CV (Parasurama et al., 2022), and simple algorithms can differentiate gender from CV with high accuracy, even after removing the most gendered data like the names, hobbies or gendered words (Parasurama and Sedoc, 2021). On the contrary, data related to personality facets seems better suited for a hiring algorithm’s training, mostly because they are less impacted by gender compared to other data traditionally used in the hiring process and are valid predictors of job performance.

Drawing upon these conclusions, our study examined the gender equity of a novel personality-based hiring algorithm. The overarching aim was to establish whether the algorithm would recommend equal numbers of males and females for several occupations; thus, not being biased toward one gender or another. As hypothesized, results demonstrate that the algorithm does not show gender inequalities when recommending the best-suited candidates for the role, meaning there is no adverse impact. In this sense, impact ratios were in the recommended standard by the EEOC for 95% of the predictive models created. Only 5% of the predictive models fell short below and are considered as having a slight impact. These results illustrate that, when they are trained with the right data, algorithms could help in building more efficient selection processes, which are also fairer for women.

From a theoretical perspective, this work improves our knowledge about how to build gender-blind hiring algorithms by using data related to personality. Also, it complements other studies, by showing that biases and adverse impacts can be reduced even when screening facet-level. Our study demonstrates that while certain distinct differences may exist between males and females concerning specific facets, these disparities become less impactful when viewed within a broader constellation of multiple facets. By aggregating these characteristics with other facets that display similarity across genders, we effectively mitigate the potential for adverse impacts. This approach ensures a more balanced and fair assessment, underscoring the fact that individual variations do not necessarily lead to gender-based discrimination when considered in a comprehensive personality algorithm framework. Ultimately, the crucial question is not about these algorithms achieving perfect fairness in their predictions. Instead, it is about determining whether they enhance existing methods and surpass the current human-driven *status quo*. While the use of algorithms does raise essential and legitimate concerns, their potential for fostering more efficient and fairer decision-making processes cannot be overlooked, especially when they are trained with appropriate data. In particular, their potential to ensure a more balanced playing field for women is a significant step forward in achieving equity. In addition, our study provides evidence that even simple algorithms can effectively reduce gender discrimination. Many individuals have expressed concerns about using algorithmic hiring processes due to a lack of understanding (Liem et al., 2018). However, our findings demonstrate that explainable algorithms can have a significant positive impact. By showcasing the potential of such algorithms, we aim to encourage the adoption of fair and unbiased decision-making tools in hiring.

Moreover, our conclusions are opening the way for future research about personality-based hiring algorithms. First, an interesting question arising from this work is about the capacity of such algorithms to be applied in practice, where they will probably be trained on male-dominated samples, as many could be forced to do due to the current disparities in the workplace. However, even if an algorithm is trained on a male-dominated sample, it could still provide fair outcomes when applied to a balanced or neutral sample, if it leverages data equally representative of both genders. This potential fairness arises from the algorithm’s reliance on well-distributed data, where the features it uses for prediction are equally prevalent in both males and females. For example, if an algorithm is trained to predict job performance based on facets like imagination, trust or self-efficacy. Although these traits might be learned from a male-dominated sample, they are not exclusive to any gender (Kajonius and Johnson, 2018). Males and females alike can exhibit high levels of imagination, trust or self-efficacy. Therefore, if the algorithm focuses on these universally applicable facets rather than gender-specific features, it should provide fair and unbiased predictions when applied to a gender-balanced sample (Kubiak et al., 2023a). Second, it is still unclear whether these kinds of algorithms could display the same results for other kinds of discrimination, for example, disability-based discrimination, which remains intense (Lippens et al., 2023). Third, even if our study showed that there was no adverse impact for 95% of the predictive models tested, we still need to address the 5% remaining: while their impact ratios are really close to the EECO requirements and do not fall lower than 0.71, some adjustments are required in order to use them in high-stakes hiring practice and be confident that they will not harm any group based on gender. For these models, future research could focus on addressing the diversity-validity dilemma, which concerns the tradeoff between selecting valid predictors of performance while minimizing adverse impact (Pyburn et al., 2008; Rupp et al., 2020; Rottman et al., 2023). As such, it seems necessary to identify strategies to target facets within the predictive model that lower the impact ratio, and to propose alternatives. It could also foster the algorithm’s explainability, by being transparent about the predictive model limitation, and how one could improve it to make it fairer regarding gender while making the smallest compromises possible about validity. For example, studies could use a feature importance framework to iteratively prune biased features with the lowest predictive power from the model.

Our work also has several practical implications. First, given the prevailing talent shortage, employers are increasingly finding it challenging to fill roles effectively. As such, it is imperative they shift focus and explore alternate indicators of potential, beyond traditional markers like academic degrees, to truly uncover and understand the essence of talent and assess the employability of their candidates (Chamorro-Premuzic, 2017). Employers can consider personality as a compelling alternative to traditional CV-based assessments, as it relates performance while being less susceptible to gender bias (Schmidt et al., 2016; Sackett et al., 2022). In addition, it is becoming increasingly imperative for employers to demonstrate that their hiring practices and tools are devoid of biases, ensuring that no particular group is unfairly disadvantaged based on their gender (Hunkenschroer and Kriebitz, 2023). Our study shows great potential in helping employers to accurately identify the underlying mechanisms of performance for a specific occupation and to reduce gender biases. That way, they might be able to hire people who are better suited for the role and perform better, and who are more diverse in terms of gender. Secondly, personality-based algorithms, by increasing the fairness of the hiring process, could probably promote organizational attractiveness. Indeed, considering the existing labor talent shortages and the significant role of an organization’s recruitment process perception in determining a candidate’s decision to accept a job offer (Hausknecht et al., 2004), enhancing the perceived fairness of algorithmic recruitment tools carries substantial implications. Recent research showed that algorithm-driven hiring processes are perceived as less fair compared to human-only decisions by candidates (Lavanchy et al., 2023) and that people feel less capable of influencing the outcome of an algorithm compared to human judgment (Li et al., 2021; Hilliard et al., 2022). Interestingly, fairness mediates the association between an algorithm-based selection process and organizational attractiveness and the intention to further proceed with the selection process (Köchling and Wehner, 2022). Consequently, it is in the best interest of employers to utilize personality-based algorithms, due to their increased fairness, to improve their attractiveness among potential candidates. This ensures that candidates are not discouraged or deterred from the process due to the perception of algorithmic unfairness. Thirdly, implementing an algorithm-based evaluation system could potentially boost the number of female applicants for a company and enhance the completion rates for the assessment process. This is due to the observed tendency of women being more inclined to complete an assessment when informed that the evaluation is conducted by an algorithm, rather than a human recruiter (Avery et al., 2023). Such a shift could play a pivotal role in fostering gender diversity within organizations by expanding the pool of female candidates applying for jobs. Heilman (1980) found that both male and female evaluators made significantly more favorable personnel decisions when females constituted 25% or more of the total candidate pool. Thus, increasing the representation of females in the candidates pool through algorithm-based evaluations could lead to more balanced hiring outcomes. Fourthly, our study serves as a useful guide for employers navigating forthcoming legislation such as New York’s AI hiring law. Recently enacted, the NYC Automated Employment Decision Tool law mandates employers using AI in hiring to disclose its use to candidates. Further, it necessitates annual independent audits to demonstrate the absence of discriminatory practices in their systems. Moreover, candidates are granted the right to request information from potential employers about what data the technology collects and analyzes. Non-compliance with these regulations could result in fines of up to $1,500. Our study helps employers align their processes with these requirements, paving the way for transparent, accountable, and unbiased algorithm-driven hiring.

## 7. Limitations

Several limitations of this research should be taken into consideration. First, while the strength of our study was that it considered 208 occupations across 21 categories, we did not include occupations that are more stereotypically judged as being gender specific. Therefore, future research can aim to retest our algorithm on an even wider array of job categories and focus specifically on occupations which are perceived to be predominately feminine. For example, studies showed that occupations related to caregiving are seen as being more feminine (Couch and Sigler, 2001), or that it persists presumptions about the gender of people employed in healthcare, notably nurses (Ekberg and Ekberg, 2017). Our sample unfortunately did not include occupations from these highly stereotypical categories. We could not include these occupations in our study, as none of the participating employers were recruiting for such roles. In fact, the employers utilizing the online platform were primarily focused on filling business-related positions (see Table 4).

Secondly, our study’s scope was limited to gender as a characteristic, which leaves room for further exploration. Recent research indicates the existence of intersectional effects between various attributes. For instance, Derous and Pepermans (2019) uncovered a “double jeopardy” situation for Maghreb/Arab female applicants applying for high-cognitive demand roles–an issue not apparent in applications for low-cognitive demand jobs. Such findings emphasize the necessity for more nuanced investigations that consider the interactions between multiple characteristics. Future research could delve into the potential adverse impacts of personality-based algorithms by examining intersectionality, such as the combined effect of gender and ethnicity. This could pave the way for more comprehensive understanding and better refinement of fair algorithmic-based hiring practices.

Third, our study tested the algorithm on males and females, as data collection for these genders was simpler and more easily accessible. However, we acknowledge that there are numerous gender non-conforming categories. Unfortunately, we did not find any satisfactory published research which studied how personality differs between males, females and people identifying as gender diverse. The only evidence we have drawn upon is the analysis proposed by Anzani et al. (2020), which delved into the personality patterns of a transgender cohort compared with normative samples of cisgender females and males. Their findings revealed that transgender women scored lower than cisgender women on two primary domains (Negative Affectivity and Psychoticism) and on seven facets. Transgender men, meanwhile, scored lower than cisgender men on Antagonism and five other facets. However, these results were derived from relatively small sample sizes of transgender individuals, all of whom were pursuing medical treatments. Consequently, these findings may not accurately represent the broader transgender and gender-non-conforming population. This indicates the necessity for future investigation into the algorithm’s gender neutrality, especially when considering the inclusion of diverse groups beyond the traditional gender binary.

Finally, we should also mention potential bias in how the rating of each employee (from 1 to 5) was made by their manager. Indeed, even though managers were prompted to reflect on the employee’s productivity and objective performance, no other specific guideline was proposed. As a result, there is a chance that different managers could have reflected upon different types of performance when making their ratings. Gender bias has also frequently been identified in performance appraisal. For example, (1) Correll et al. (2020) showed that it exists differences in the language used to describe females and males performance and that the same behaviors could impact performance ratings differently depending on the employee’s gender, (2) Benson et al. (unpublished) revealed differences in potential ratings between gender, and, (3) Rivera and Tilcsik (2019) showed that the number of scale points used for the evaluations significantly affect the size of the gender gap in male-dominated fields. Still, there are reasons to believe that the ratings made were accurate estimates of objective performance: (1) as shown by Jackson and Furnham (2001), biases such as halo do not necessarily reduce rating accuracy, and supervisor ratings are useful measures of overall performance, (2) managerial ratings have a good corrected mean correlation with objective performance for salesperson job performance (Jaramillo et al., 2005), which is a type of role composing one-third of our total sample, (3) ratings have been shown to be more accurate for unskilled, skilled and professional workers compared to managerial occupations (Miller and Thornton, 2006), and these three levels of occupations are the most represented in our sample, and (4) each of the scale’s point were clearly defined in the rating form. Other studies should, however, try to measure performance in a more structured and controlled manner. Furthermore, future research should also incorporate a more comprehensive understanding of job performance, considering a wide range of relevant factors. For instance, Rotundo and Sackett (2002) pinpointed three broad components of job performance: task performance, citizenship behavior, and counterproductive performance. They further demonstrated that two primary elements of performance–tasks performance and counterproductive performance–were the more weighted by raters. Recent research also suggests an increasing interest in other types of performance. Contextual performance, for example, includes behaviors that contribute to the social and psychological environment (Ramos-Villagrasa et al., 2022). Adaptive performance, on the other hand, pertains to an employee’s ability to modify their thoughts, behaviors, and emotions to adapt to their evolving work environment. Such adaptations can encompass adjustments to new technologies, procedures, business processes, or work roles (Baard et al., 2014). Given that meta-analyses have revealed that traits have differential relationships with contextual (He et al., 2019) and adaptive performance (Huang et al., 2014), it would be prudent to incorporate these insights in future research.

## 8. Conclusion

Gender stereotypes are incredibly stable. For example, Offermann and Coats (2018) showed that ILTs (Implicit Leadership Theories) did not change during the last 20 years, despite organizational and societal changes. Also, large-scale cross-national field experiments highlight occupational gender composition (Birkelund et al., 2022; Adamovic and Leibbrandt, 2023), showing disparate proportions of individuals of a particular gender working in specific occupations. This is particularly salient in online hiring, which triggers the use of cognitive shortcuts about the role-specific abilities of each gender (Galperin, 2021). This persistence of gender discrimination in hiring, despite all the efforts made for so many years, calls for the identification of strategies that will lead to an effective and lasting response. The findings from our research suggest that personality-based hiring algorithms serve as an effective solution, demonstrating non-adverse impact in most instances. In other words, they do not unfairly disadvantage certain groups of people based on their gender. Properly trained and used, these algorithms could help organizations to build fairer decision-making processes.

## Data availability statement

The datasets presented in this article are not readily available because even if the data used has been anonymized by AssessFirst, the participants may choose to delete their data at any time: while the data were accessible for scientific purposes during the analysis and publication, it cannot be ensured that the same data will remain legally available in the future. For legal reasons supporting data is, therefore, not available. Requests to access the datasets should be directed to EK, ekubiak@assessfirst.com.

## Ethics statement

Ethical review and approval was not required for the study on human participants in accordance with the local legislation and institutional requirements. The studies were conducted in accordance with the local legislation and institutional requirements. Written informed consent for participation was not required from the participants or the participants’ legal guardians/next of kin in accordance with the national legislation and institutional requirements because written informed consent from the participants was not required to participate in this study in accordance with the national legislation and the institutional requirements.

## Author contributions

EK and SB: conceptualization, methodology, data collection, and data analysis. EK: supervision. EK and ME: writing—original draft. All authors contributed to the writing—review and editing and read and agreed to the current version of the manuscript.
